# AAV vector-meditated expression of HLA-G reduces injury-induced corneal vascularization, immune cell infiltration, and fibrosis

**DOI:** 10.1038/s41598-017-18002-9

**Published:** 2017-12-19

**Authors:** Matthew L. Hirsch, Laura M. Conatser, Sara M. Smith, Jacklyn H. Salmon, Jerry Wu, Nicholas E. Buglak, Rich Davis, Brian C. Gilger

**Affiliations:** 1 Gene Therapy Center, University of North Carolina at Chapel Hill, Chapel Hill, NC 27599 USA; 2 Department of Ophthalmology, University of North Carolina, Chapel Hill, NC 27599 USA; 3 Department of Clinical Sciences, North Carolina State University, Raleigh, NC 27607 USA

## Abstract

Over 1.5 million individuals suffer from cornea vascularization due to genetic and/or environmental factors, compromising visual acuity and often resulting in blindness. Current treatments of corneal vascularization are limited in efficacy and elicit undesirable effects including, ironically, vision loss. To develop a safe and effective therapy for corneal vascularization, adeno-associated virus (AAV) gene therapy, exploiting a natural immune tolerance mechanism induced by human leukocyte antigen G (HLA-G), was investigated. Self-complementary AAV cassettes containing codon optimized HLA-G1 (transmembrane) or HLA-G5 (soluble) isoforms were validated *in vitro*. Then, following a corneal intrastromal injection, AAV vector transduction kinetics, using a chimeric AAV capsid, were determined in rabbits. One week following corneal trauma, a single intrastromal injection of scAAV8G9-optHLA-G1 + G5 prevented corneal vascularization, inhibited trauma-induced T-lymphocyte infiltration (some of which were CD8^+^), and dramatically reduced myofibroblast formation compared to control treated eyes. Biodistribution analyses suggested AAV vectors persisted only in the trauma-induced corneas; however, a neutralizing antibody response to the vector capsid was observed inconsistently. The collective data demonstrate the clinical potential of scAAV8G9-optHLA-G to safely and effectively treat corneal vascularization and inhibit fibrosis while alluding to broader roles in ocular surface immunity and allogenic organ transplantation.

## Introduction

The normal cornea is considered to be an immune privileged site that has evolved tolerance to ocular surface flora and environmental irritants in an attempt to prevent overt ocular inflammation that can result in blindness. This immune suppressed status is maintained in part by the absence of blood or lymphatic vessels, as well as a lack of MHC class II molecules on most of the cells that form the cornea^1,2^. A broad range of environmental insults and genetic conditions manifest in the form of corneal vascularization which occurs at a remarkably high rate of incidence, approximately 1.4 million patients per year in the Unites States alone^3^. In addition to visual disturbances from opacities associated with corneal vascularization, the loss of immune tolerance in the vascularized cornea disrupts the normal immunologic privilege, which leads to increased inflammation and scarring, and is a leading cause of cornea transplant rejection^4,5^. Medical options for the treatment of cornea vascularization, such as anti-inflammatory drugs, demonstrates limited and/or varied success along with the potential development of common vision-threatening side-effects including cataracts and glaucoma. Long-term use of these treatments, in addition to the requirement for frequently repeated daily applications leads to poor patient compliance and therefore, remains to be a significant concern. Additionally, if the drug regimens fail, several surgical options for treating cornea vascularization are considered, however, they can also induce neovascularization.

Gene delivery approaches using adeno-associated virus (AAV) vectors are currently the leading method of human gene therapy and have been used in hundreds of clinical trials for over 20 different diseases (clinicaltrials.gov)^6^. In regard to AAV gene therapy for the treatment of corneal neovascularization, work nearly a decade ago demonstrated that subconjunctival injections of AAV serotype 2 (AAV2) vectors harboring angiostatin or endostatin cDNA prior to cornea trauma, reduced neovascularization in rodents^7,8^. These reports provide an important proof of concept for using AAV gene therapy as a prophylactic for cornea vascularization. However, in these examples the effects were incomplete, evaluated only over a short term (2 weeks), and did not demonstrate efficacy in the perceived clinical situation, administration following a cornea trauma. Additionally, these approaches overlook the complex pathogenesis of corneal diseases by addressing only one symptom, corneal neovascularization, while reinstating corneal immune homeostasis, hypothetically, should prevent the onset and/or cause regression of vascularization and many of the causes there of.

The immunomodulatory and anti-inflammatory molecule, human leukocyte antigen-G (HLA-G) was originally discovered in the human placenta where it plays a role in preventing fetus rejection by the maternal immune system, a process termed fetal-maternal tolerance^9–11^. Seven different HLA-G isoforms have been described with the transmembrane, or exosome-associated, and soluble isoforms, HLA-G1 and HLA-G5, respectively, among the most studied^12^. Generally, the immune suppression effects of HLA-G are elicited directly, via binding of immune cells to HLA-G1 and the subsequent induction of apoptosis, or indirectly, by preventing vasculature formation and inducing endothelial cell apoptosis (perhaps the major role of HLA-G5)^12^. Additionally, HLA-G induces toleragenic dendritic cells, upregulates T regulatory cells (including CD4^+^ HLA-G^+^), and inhibits reactive oxygen species production and phagocytosis by neutrophils^13–16^. Low levels of HLA-G are associated with a higher incidence of graft versus host disease, while higher levels of HLA-G are consistent with reduced transplant rejection^17^. For instance, HLA-G delayed skin allograft rejection in a murine model^18^.

The purpose of this study was to engineer and validate a therapeutic, based on a natural mechanism of immune suppression, for the treatment of corneal vascularization following an insult. Towards this end, HLA-G cDNA was enhanced by codon optimization and validated in a self-complementary AAV vector context *in vitro* ^19,20^. Then, the optimized HLA-G1 and HLA-G5 cassettes were packaged in a capsid proficient for multi-species cornea gene delivery^21^ and administered via a single intrastromal injection, 7 days following a corneal chemical burn in a rabbit model. In contrast to the control injected corneas, which became heavily vascularized, corneas that received scAAV8G9-optHLA-G demonstrated the near complete absence of vasculature over a 2-month period following the insult. The results also demonstrated decreased immune cell infiltration of the cornea, which included cytotoxic T lymphocytes, and decreased smooth muscle actin staining in the scAAV8G9-optHLA-G treated corneas. Biodistribution analysis following an intrastromal injection in traumatized corneas showed that, although vector genomes were not detected outside of the cornea, an AAV capsid neutralizing antibody response was elicited in approximately 50% of the cases. The collective data demonstrate the clinical potential of scAAV8G9-optHLA-G to eliminate vascularization of the cornea while alluding to even broader roles in fibrosis, ocular surface immunity, and allogenic organ transplantation.

## Results

### Optimized AAV HLA-G construct and *in vitro* expression

**Figure 1:**
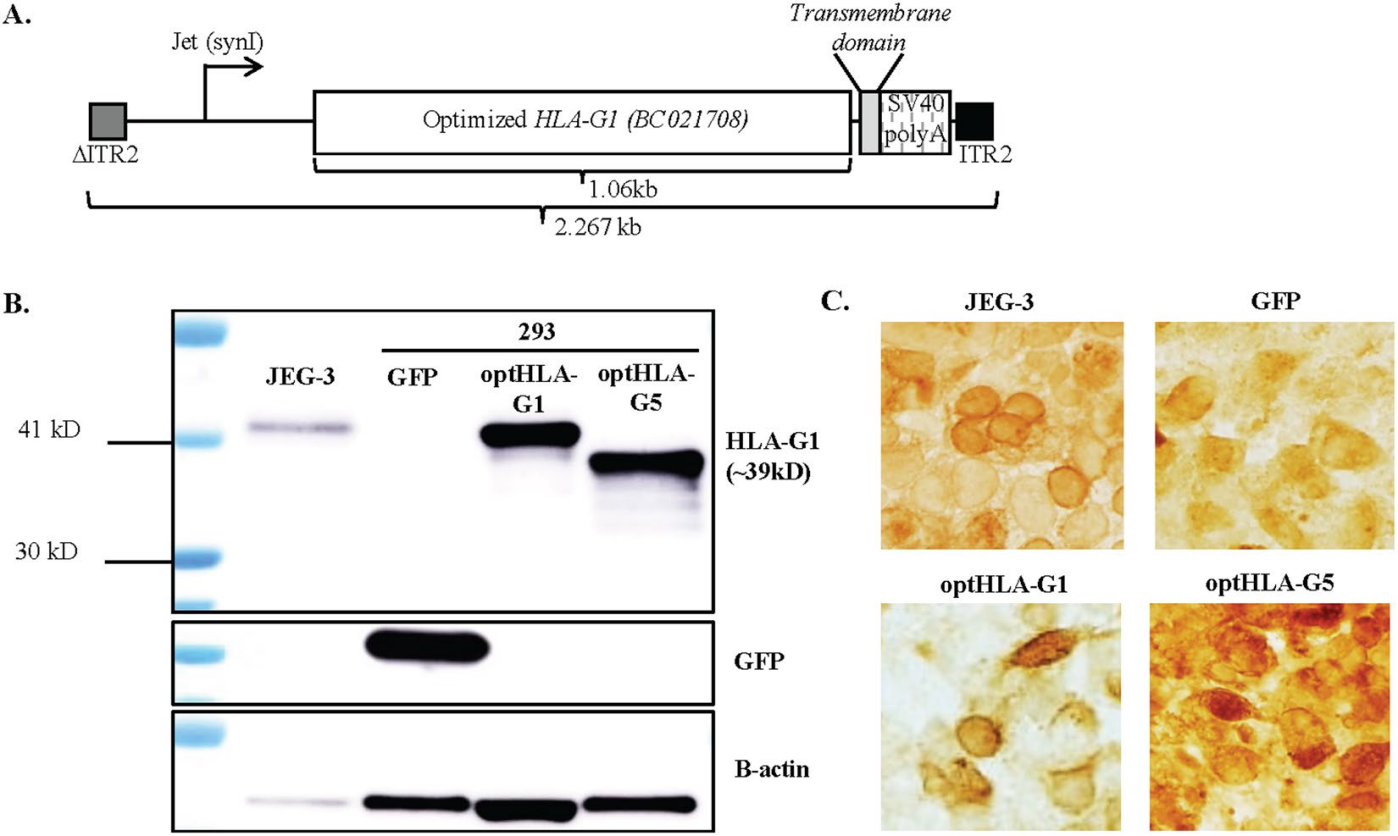
Characterization of scAAV8G9-optHLA-G *in vitro*. (**A**) A cartoon of the self-complementary scAAV8G9-optHLA-G vector cassette. (**B**) Confirmation of optHLA-G isoform production in 293 cells by Western blot. JEG-3 cells are placental cells and serve as a positive control. To concisely represent the data, blot images have been cropped and aligned while images of full-length blots are shown in Supplementary Figure 4. (**C**) Diaminobenzidine staining of optHLA-G isoforms, imaged at the same exposure times, following transfection of 293 cells with the indicated cDNAs. ITR = inverted terminal repeat.

Initially the HLA-G1 cDNA (accession BC021708) was codon optimized for theoretically enhanced transgene production in human cells, as well as to remove unwanted alternative open reading frames (ORFs) (Fig. 1A)^22^. Western blot detection of plasmid-borne HLA-G1 from either WT or codon optimized HLA-G1 cDNA demonstrated a consistent 3-fold elevation when using the codon-optimized version (optHLA-G1) in human embryonic 293 cells (Fig. S1). Next, the optHLA-G1 transmembrane domain was deleted to generate optHLA-G5 cDNA, and both optHLA-G1 and optHLA-G5 were cloned into a self-complementary AAV plasmid context (Fig. 1A). Western blot analysis following transfections of these plasmids in 293 cells generated an anticipated single band at 39 kDA for optHLA-G1 and the expected smaller product for optHLA-G5 (Fig. 1B). Next, the intracellular localization of HLA-G1 (transmembrane) and HLA-G5 (soluble and secreted) were investigated via immunocytochemistry following transfections in 293 cells. Immunostaining of placental JEG-3 cells demonstrated cytoplasmic, surface, and extracellular staining (Fig. 1C). In contrast, 293 cells transfected with optHLA-G1 demonstrated primarily surface membrane staining while optHLA-G5 transfected cells appear to have more staining in the cytosol and extracellular matrix (Fig. 1C). These collective results demonstrate that the optimized HLA-G isoforms result in efficient protein production and the expected cellular localization.

### AAV8G9 capsid mediates efficient gene delivery to the rabbit cornea

**Figure 2:**
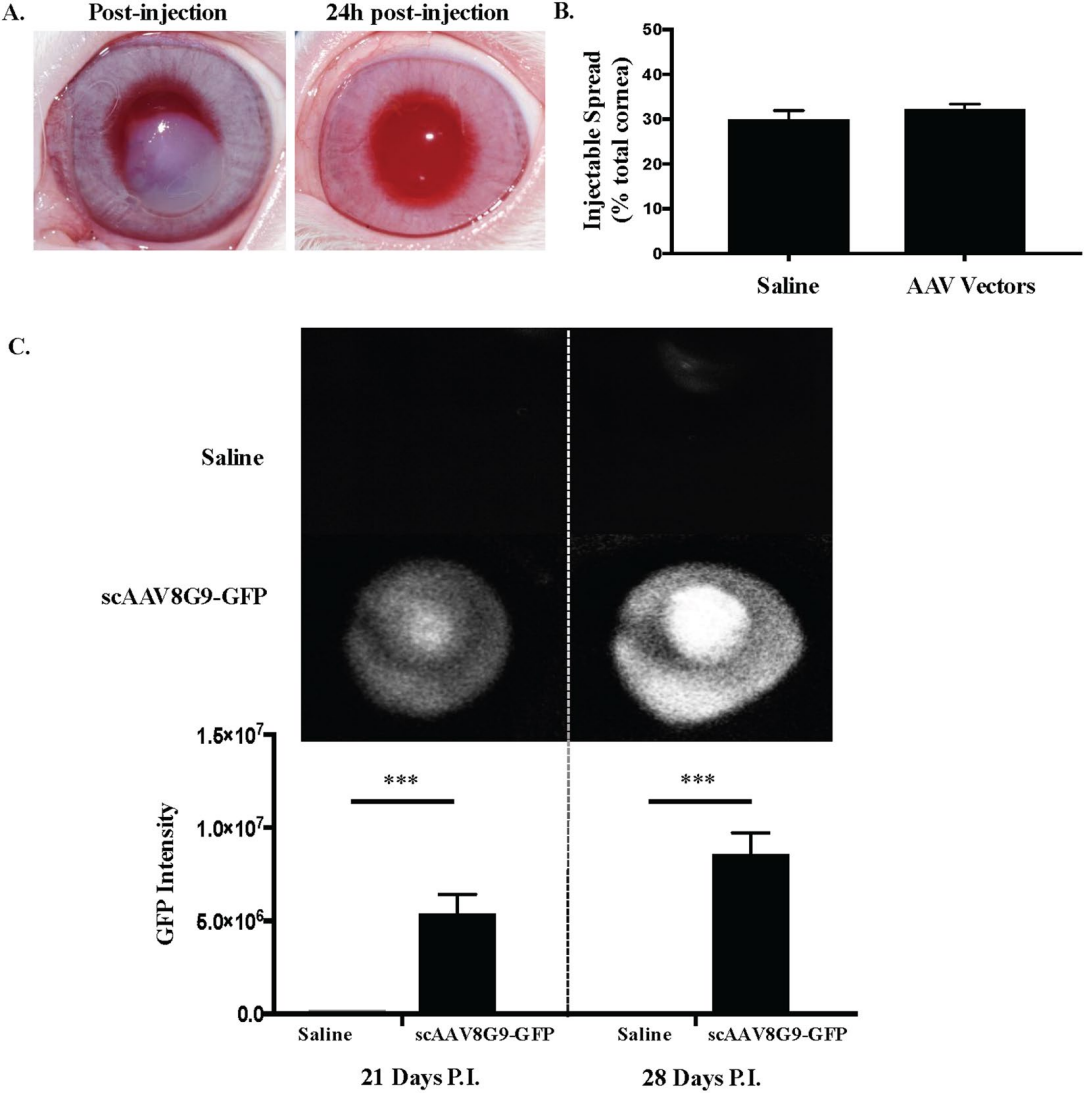
scAAV8G9 Mediates Gene Delivery in the Rabbit Cornea. **A**) Vector injection of 50ul into the rabbit corneal stroma causes transient corneal cloudiness. **B**) Quantitation of the area of cornea covered by the injection. **C**) Representative *in vivo* images depicting intensity of GFP expression and quantitation of GFP intensity of the indicted injectable at the indicated time point. (p < 0.0009). P.I. = post-injection, sc = self-complementary.

The chimeric capsid “8G9”, which is the AAV8 capsid sequence engrafted with the putative AAV9 galactose receptor binding domain, was found superior for human cornea transduction following intrastromal injection^21^. To determine if AAV8G9 also transduces the rabbit cornea, a self-complementary (sc) JET-GFP reporter cassette was packaged in the AAV8G9 capsid and administered to the rabbit cornea via a 50 µl intrastromal injection of 5 × 10^10^ viral genomes (vg)^23^. Following an intrastromal injection of balanced salt solution (BSS) or scAAV8G9-GFP, an immediate central corneal opacity throughout approximately 30% of the central cornea developed (Fig. 2A,B). This opacity completely resolved within 24 hours of the injection (Fig. 2A). Using live imaging, GFP fluorescence was detected in the central cornea, at the location of the vector injection, twenty-one days post-injection (Fig. 2C). Quantitation of the signal demonstrated peak GFP intensity at 28 days post-injection (Fig. 2C).

### Effect of scAAV8G9-optHLA-G on corneal vascularization *in vivo*

**Figure 3:**
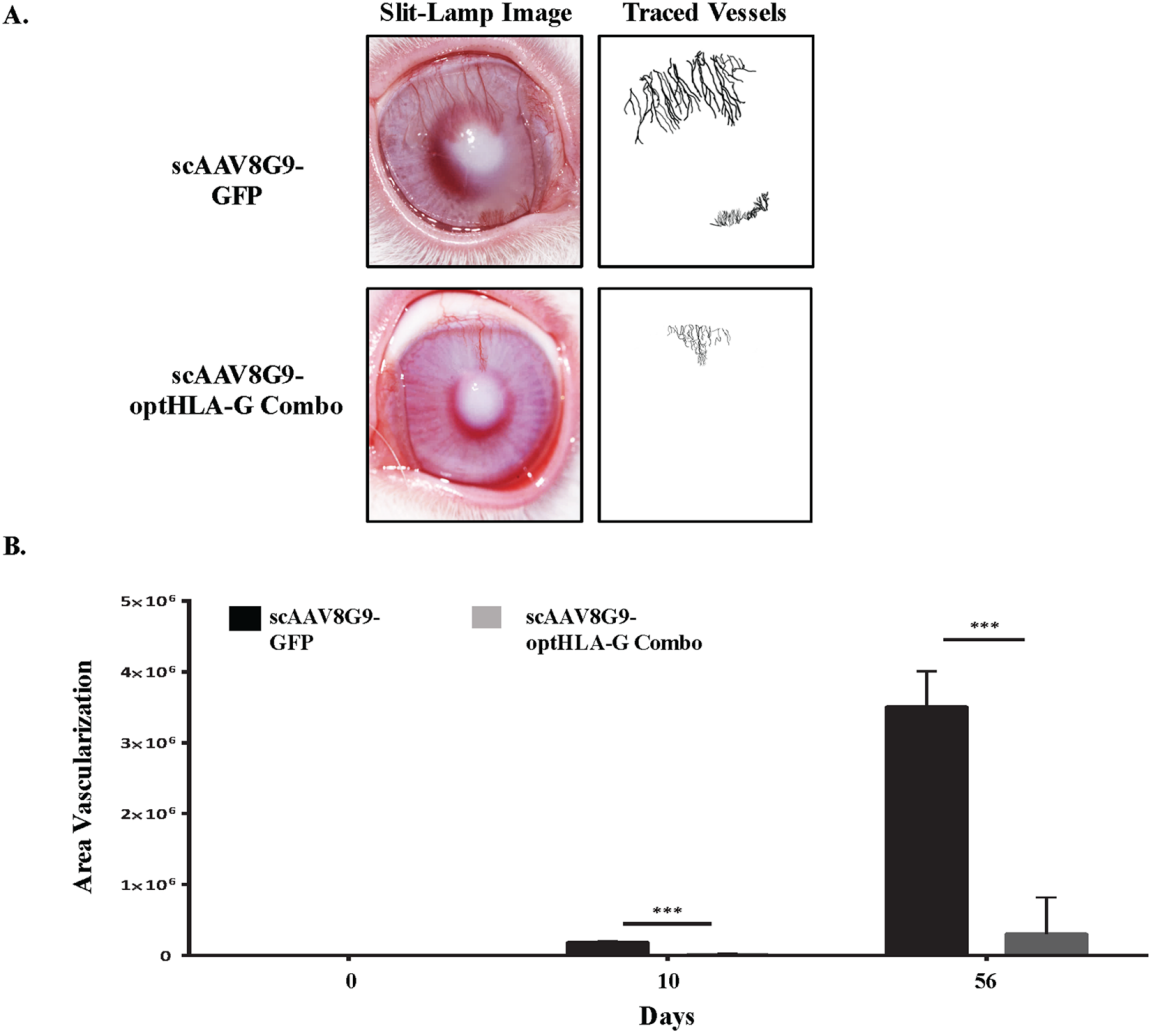
scAAV8G9-optHLA-G Combo Prevents Burn-induced Cornea Vascularization. Rabbit corneas were centrally burned seven days prior to a cornea intrastromal injection of scAAV8G9-GFP or scAAV8G9-optHLA-G Combo (isoforms 1 and 5 at a 1:1 ratio). (**A**) Representative *in vivo* images depicting vascularization alongside images of vascularization tracings of treated corneas 42 days post-injection with the indicated vectors. (**B**) Quantitation of the traced area of vascularization immediately after corneal burn (day 0), 3 days after injection with the indicated vectors (day 10), and the last experimental time point (day 56) (p < 0.001 at day 10 and p < 0.002 at day 56). sc = self-complementary.

A previous report using HLA-G protein demonstrated HLA-G5′s function, consistent with its known roles in humans, in a rabbit model^24^. Additionally, the rabbit eye is similar in structure and size to the human eye making it a popular model for ocular research. Given that the AAV8G9 capsid was validated for rabbit cornea transduction (Fig. 2), the optHLA-G plasmid constructs were used to produce preclinical scAAV8G9 vectors. To mimic a clinically relevant scenario, rabbits were anesthetized and corneas were injured, prior to AAV vector injections via a 5 mm central corneal chemical burn with NaOH. Seven days after burn injury, intrastromal injections of scAAV8G9 vectors encoding GFP or, due to the exact roles of HLA-G isoforms being unknown and in an effort to increase the chances of seeing a therapeutic effect, optHLA-G1 + optHLA-G5 at a 1:1 ratio (optHLA-G Combo). There were no significant changes associated with intraocular pressure or corneal thickness by 24 hours after injection and throughout the experimental evaluation period (Fig. S2). Corneal vascularization began approximately 7 days after injury, with a significant difference in vascularization between treatment groups by day 10 post-injury (*p* < 0.001) (Fig. 3). Quantitation of the area of vascularization using ImageJ software demonstrated increased vessel in-growth at every experimental time point, with maximal vascularization, in corneas treated with scAAV8G9-GFP, at 56 days post-injury. In contrast, 2 months post-injury, subjects that received scAAV8G9-optHLA-G Combo did not manifest any significant vessel formation, which is 10-fold less than the scAAV8G9-GFP treated eyes (*p* < 0.002) (Figs 3 and S3).

### HLA-G production and immune inhibition in the injured cornea

**Figure 4:**
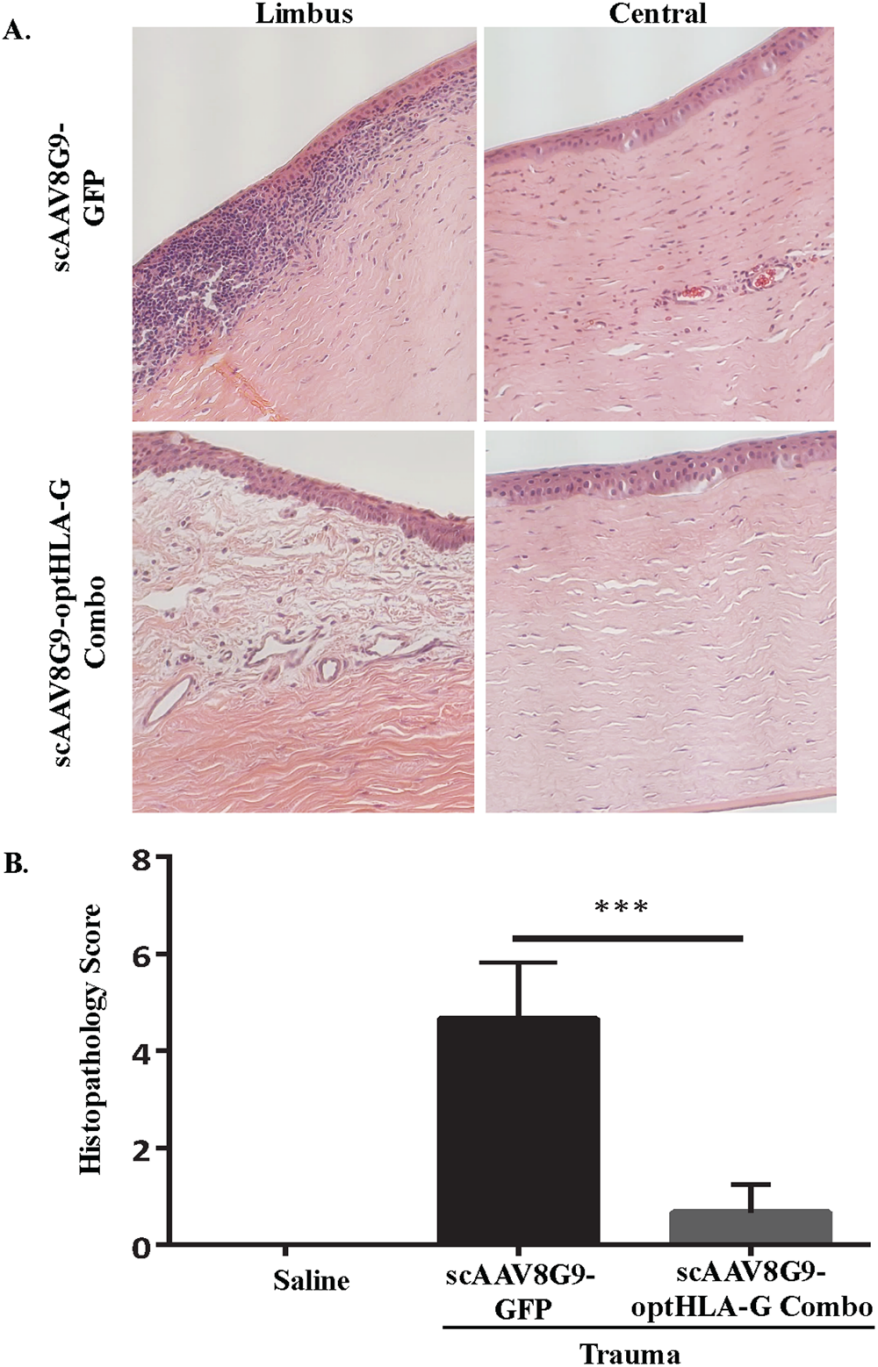
scAAV8G9-optHLA-G Combo Prevents Cornea Burn-induced Vascularization and Immune Cell Infiltration. Rabbit cornea sections were acquired 60 days following the injection of indicated vectors into trauma induced corneas and stained with hematoxylin and eosin. (**A**) Representative images of processed sections treated with the indicated vector preparation. (**B**) Quantification of clinical histological exam scores of all H&E sections presented in (**A**) (p < 0.0005).

Following 8 weeks of observation, tissues were recovered for analysis. Histology of injury induced corneas injected with scAAV8G9-GFP revealed changes consisting of central corneal mononuclear cellular infiltrate, vascularization into the central cornea, and mild to moderate amount of fibrosis (Fig. 4A). While in comparison, histological analysis of injured corneas that were injected with scAAV8G9-optHLA-G Combo, had minimal cellular infiltrates and no vascularization (Figs 4B, S4A). In fact, the mean cumulative histology scores of post-injury corneas injected with scAAV8G9-HLA-G Combo were significantly lower than the mean scores for corneas injected with the GFP control vector (*p* < 0.0005) (Figs 4B, S4A).

**Figure 5:**
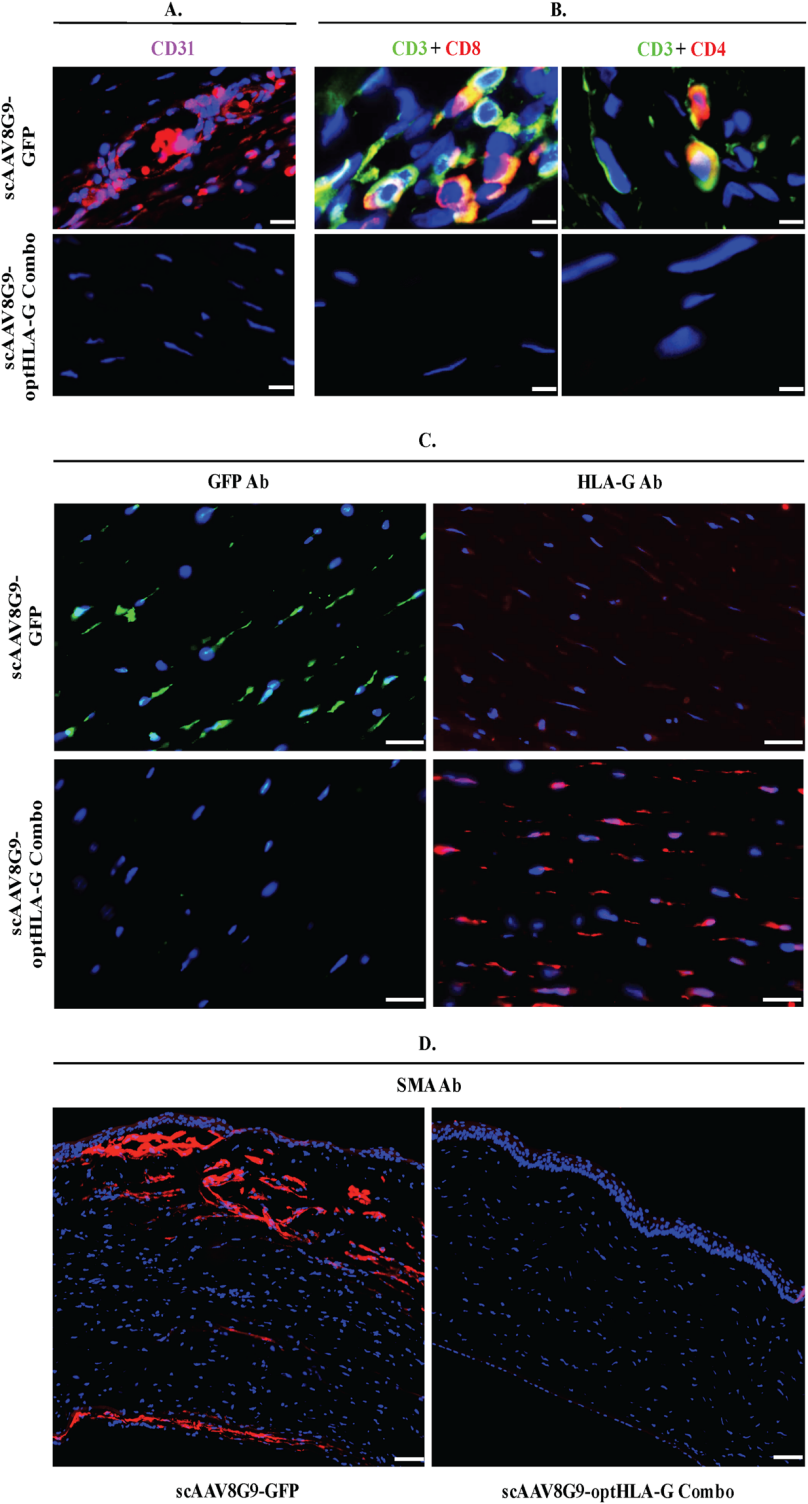
scAAV8G9-optHLA-G Combo Prevents Cornea Burn-induced Vascularization and Cytotoxic T-cell Infiltration. Rabbit cornea sections were acquired 60 days following the injection of indicated vectors into burn corneas and stained for an endothelial cell marker (CD31), T cell markers, transgene abundance, and αSMA in the indicated treatment groups. Scale bars = (**A**) 10 µm, (**B**) 5 µm, (**C**) 20 µm, (**D**) 200 µm.

In addition to H&E staining, immunofluorescence was used to confirm blood vessels in the central cornea by revealing the presence of the endothelial intercellular junction cell marker, CD31 (Fig. 5A). Immunostaining for CD3 confirmed that the majority of infiltrating cells in the injured corneas treated with scAAV8G9-GFP were positive for the CD3 surface antigen (Fig. 5B). A subset of this population also stained positive for CD8, demonstrating the influx of cytotoxic T cells following cornea injury (Fig. 5B). A smaller proportion of this T cell population proved to be CD4^+^, thereby demonstrating the presence of helper T cells.

Next, cornea tissue was analyzed to correlate the phenotypic outcome to vector derived transgene production using immunofluorescence. Similar to our reported human results^21^, GFP was present throughout the cornea of rabbits given scAAV8G9-GFP (Fig. 5C). As expected, HLA-G proteins were evident only in corneas injected with scAAV8G9-optHLA-G Combo, thereby correlating the therapeutic effect to HLA-G (Fig. 5C). Furthermore, immunofluorescence of injured corneas treated with optHLA-G Combo vectors were negative for alpha smooth muscle actin (αSMA) while those treated with the GFP control vector demonstrated a positive signal throughout the total thickness of the cornea, with the strongest effect seen in the anterior portion of the stroma and along the endothelium (Fig. 5D). In addition to the difference in αSMA expression, a disruption to the organization of the cells and/or the collagen fibrils in the scAAV8G9-GFP treated tissues was observed (Figs 5D, S4B), suggesting that HLA-G may play a role in preventing corneal fibrosis.

### AAV8G9 Biodistribution following Intrastromal Injection of Traumatized Corneas

It is of importance for potential clinical applications to determine the biodistribution of persistent AAV vectors following scAAV8G9 intrastromal injections. To do this, total DNA was recovered from samples of liver and two brain regions; the trigeminal nerve nuclei, that innervates the cornea, and the lateral geniculate nucleus that innervates the retina. PCR using primers to the transgenic cassette failed to detect any viral genomes outside of the cornea. Despite this, at the conclusion of the study, a strong scAAV8G9 capsid-specific neutralizing antibody response was detected in one of the rabbits that received scAAV8G9-GFP treatment and had developed extensive corneal vascularization, with a titer of 1/256 (Table 1). Two other rabbits demonstrated a very mild antibody response to the scAAV8G9 capsid, independent of the transgene or the emergence of vascularization (Table 1).

**Table 1:** Neutralizing Antibody Titer Following scAAV8G9 Intrastromal Injection of Burned Corneas.

Animal ID	Serum Dilution
**Saline**	
Rabbit-1	no Ab
**scAAV8G9-GFP**	
Rabbit-6	1:2
Rabbit-7	No Ab
Rabbit-8	1:256
**scAAV8G9-optHLA-G Combo**	
Rabbit-12	1:4
Rabbit-13	No Ab
Rabbit-14	No Ab

## Discussion

Corneal vascularization, which affects over 1.4 million people per year in the United States alone, is elicited by one of two kinds of conditions, inflammation or hypoxia. Inflammation and/or hypoxia can be the result of a variety of insults including, bacterial or viral infection, autoimmune or degenerative diseases, trauma/manipulation, extended wear of contact lenses, and chemical burns or toxic contamination. The invasion of vessels originating in the limbus not only affects visual acuity or occludes vision but also disrupts corneal immune homeostasis resulting in secondary conditions such as, increased inflammation and scarring, and is a major component in development of dry eye and in corneal transplant rejection. Current modalities to treat vascularization rely on frequent drug administration that are only partially effective with serious vision threatening side effects. More invasive procedures such as argon laser photocoagulation and transection are not particularly successful and are increasingly less employed. Therefore, a single dose therapeutic capable of preventing, or inducing regression of vasculature, without vision threatening side effects is needed to replace the current corneal vascularization treatment deficits. Towards this end, AAV gene delivery of codon optimized HLA-G isoforms was employed post-insult in rabbit corneas. Although incompletely understood, the effects of HLA-G are multi-factorial and perhaps natural in the eye, as HLA-G has been hypothesized to play a role in ocular immune privilege^12^. Furthermore, its inherent function in fetal maternal immune tolerance implies that in humans, HLA-G is a “self” protein, an attribute important for successful gene therapy. Following *in vitro* characterization and vector validation of scAAV8G9-optHLA-G (Figs 1, 2), the experimental data demonstrate, with remarkable significance, three important therapeutic outcomes: i) near complete inhibition of corneal vascularization when administered post-trauma (Figs 3, S3), ii) maintenance of immune homeostasis by prevention of immune cell infiltration of the cornea (Figs 4, S2), and iii) decreased myofibroblast formation in the injured cornea (Figs 4 and S4). Further characterization for anticipated clinical applications indicated that AAV vectors injected into the injured corneal stroma inconsistently elicited an antibody response to the viral capsid (Table 1). However, following intrastromal injection of AAV8G9 at the indicated dose, transgenic genomes were detected outside of the cornea in the tested tissues (data not shown). Collectively, these preclinical results using scAAV8G9-optHLA-G gene therapy may facilitate the development of a single dose therapeutic capable of safely treating cornea vascularization and perhaps other ocular and non-ocular diseases.

Initially, the codon usage of HLA-G1 was altered for envisioned human applications in a manner that increased overall abundance, compared to WT HLA-G cDNA, and to eliminate alternative ORFs, which can elicit CTLs following systemic gene therapy (Fig. S1)^22^. Although codon optimization is inconsistently successful^25,26^, in the case of HLA-G1 the results demonstrate 3-fold increased abundance using the synthetic ORF, which could be due, in part, to altered post-transcriptional regulation by microRNAs targeting WT HLA-G^27,28^. Conceptually, this allows a concomitant dose decrease and, based on the available AAV vector results in humans, lower administered doses result in less vector related immune complications and better therapeutic outcomes^6^.

scAAV8G9-optHLA-G intrastromal injections were well tolerated with a near complete clearing of the cornea by 24 hours and resolution of injection-related inflammation by 3 days post-injection (Fig. 2). These injections resulted in widespread corneal transduction of GFP or HLA-G isoforms in normal and injured corneas of rabbits, a species with a cornea of similar size to human corneas (Fig. 5). Of particular note is that the injections resulted in approximately 30% corneal coverage following a total injection volume of 50 ul (Fig. 2A). This volume injected intrastromally, inconsistently elicited neutralizing antibodies to the 8G9 capsid in the serum, 2 months post-injection. This result may be related to subtle differences during the actual administration (i.e. depth of needle in the cornea) and/or perhaps to the severity of the individual corneal response to the trauma induced a week prior (i.e. proximity of neovascularization to the actual injection). In contrast, no AAV vector genomes were noted in off-target tissue (e.g., liver, brain) suggesting that either the level of peripheral virus was below the sensitive detection of PCR, or AAV vector transduction was nearly entirely restricted to the cornea (data not shown). Either way, and although not envisioned or anticipated, successful repeated AAV vector administration to the cornea has been reported independent of the AAV capsid vaccination^29^.

At the therapeutic level, when compared to scAAV8G9-GFP, corneas injected with scAAV8G9-optHLA-G Combo had significantly less vascularization and inflammatory cell infiltrate 2 months following corneal injury. Correlating to other models where HLA-G suppresses angiogenesis, T cells, NK cells, B cells, and monocyte infiltrate, these data in the rabbit cornea support the notion that AAV HLA-G therapy provides sustained immune tolerance to the ocular surface. However, HLA-G’s immune suppression capability does not come without concern, as it has been implicated in immune evasion of both solid and liquid tumors (reviewed in^12^). Therefore, in regard to HLA-G cornea gene therapy for vascularization and other anterior eye diseases, HLA-G production should be restricted minimally by a cornea specific promoter, such as keratocan, and perhaps by engrafted miRNA targets that reported to enhance tissue specific production^30,31^. The study herein, the constitutive and ubiquitous JET promoter was used^32^. Although indirect antibody evidence suggested at some point scAAV8G9 vectors leached out of the cornea in about half of the treated rabbits, we did not see any tumors develop nor did we observe any non-ocular pathologies (Table 1).

A somewhat unexpected finding was that scAAV8G9-optHLA-G Combo inhibited αSMA expression in the injured region of the rabbit cornea. αSMA is the most commonly used marker of myofibroblasts, which are induced by injury and are central to the development of corneal opacity. Upon cornea damage, cytokines including transforming growth factor beta, platelet-derived growth factor and interleukin-1 induce keratocyte differentiation to myofibroblasts, which restores the structural integrity of the cornea. However, this process inherently results in corneal fibrosis and opacity within 4 weeks in rabbits^33^. The experiments herein depict similar results in which the initial injury induced a corneal opacity throughout the experimental duration. Although the clinical corneal opacity scores were not significantly different between GFP control and scAAV8G9-optHLA-G Combo groups, the absence of myofibroblast formation supported by Masson trichrome staining results suggests HLA-G inhibits cornea fibrosis (Figs 5, S4). To our knowledge, this is among the first reports of a correlation between HLA-G and decreased fibrosis, however, in the liver of patients with chronic hepatitis B there was a significant correlation between HLA-G^+^ cells and the fibrotic area^34^. Whether HLA-G’s influence on myofibroblast formation is distinct or causally linked to the vascularization and immune inhibition cascade remains to be elucidated.

The results herein demonstrate the first application, to our knowledge, using AAV vectors for HLA-G gene therapy in any tissue, with optimism generated for a single dose safe and effective treatment for, minimally, corneal vascularization. This effectiveness of HLA-G gene therapy in cornea has widespread implications for the treatment of ocular surface disorders in which normal immunologic tolerance is disrupted, including corneal injuries, infections, immune-mediated diseases, and prevention of corneal transplant rejection in both low and high-risk patients. Beyond the cornea, the implications of scAAV-optHLA-G for the treatment of dry eye, corneal scarring, neovascular glaucoma, uveitis, retinal/choroid vascularization, and theoretically, any solid organ transplant have yet to be realized.

## Materials and Methods

### Optimized AAV HLA-G construct and *in vitro* expression

The HLA-G1 cDNA was codon optimized (GenScript USA, Piscataway, NJ) and Western blot detection was used to compare the amount of plasmid-borne HLA-G1 from either WT or codon optimized HLA-G1 cDNA. Using PCR amplification, the transmembrane domain of HLA-G1 was then deleted to generate the soluble isoform, HLA-G5, cDNA. Following transfections of these plasmids in Human embryonic kidney 293 cells, cell lysate was made and Western blot analysis was used to detect the size of the protein products that were generated. Immunocytochemistry, with the use of 3,3′-Diaminobenzidine (DAB), was used to look at the intracellular localization of HLA-G1 and HLA-G5 in transfected 293 cells. Human placenta cells (JEG-3 cells) were used as a positive control (ATCC^®^ HTB36™). Both HLA-G1 and HLA-G5 were then cloned into a self-complementary AAV plasmid context.

### *In vivo* HLA-G expression following intrastromal injection of scAAV8G9-optHLA-G

All of the animals used during this study were done so in accordance with the NIH Guide for the Care and Use of Laboratory Animals and an approved protocol monitored by the North Carolina State University Institutional Animal Use and Care committee (IACUC). Normal outbred male New Zealand White rabbits (*Oryctolagus cuniculus*) were used. While the rabbits were under anesthesia and following the application of a topical anesthetic (0.5% proparacaine HCL [Alcaine, Alcon Laboratories, Fort Worth, TX]), a unilateral corneal wound was created by incubating a 5 mm circular filter disc in 20ul of 1 M NaOH and applying it to the central cornea for 30 seconds^35,36^. Seven days after corneal wounding, each rabbit was anesthetized and both eyes received an intrastromal injection with either saline solution (balanced salt solution (BSS), Alcon Laboratories, Fort Worth, TX), or scAAV8G9 vectors encoding for GFP or, at a 1:1 ratio, a combination of HLA-G1 + HLA-G5 vectors (n = 3 for each treatment group). For each injection, a 31-gauge needle was used to deliver 50 µL of either the saline solution or, at a viral concentration of 1 × 10^9^ vg/µl, the AAV vectors into the superficial corneal stromal. Following wounding and again after intrastromal injections, prophylactic antibiotics (Moxifloxacin, Alcon Laboratories, Fort Worth, TX) were applied topically to each cornea and buprenorphine was given subcutaneously to help reduce post-operative discomfort. Post injection and while conscious, *in vivo* expression of GFP was evaluated in rabbits that were injected with scAAV8G9-GFP using a scanning laser ophthalmoscope (SLO) with a 482 nm laser source (Infrared cSLO; RetiMap Roland Consult, Germany, Wiesbaden, Germany). The intensity of GFP fluorescence expression was then quantitated from the *in vivo* images using a previously described method for calculating corrected total cell fluorescence (CTCF), where CTCF is equal to, Integrated Density - (Area of selected cell/ fluorescence X Mean fluorescence of background readings)^37,38^.

### Effect of scAAV8G9-optHLA-G on corneal vascularization *in vivo*

Neovascularization was monitored by slit-lamp biomicroscopy and photography, central corneal pachymetry (PachPen Handheld Pachymeter, Accutome, Malvern, PA), and intraocular pressure (IOP; Tonovet tonometer, Icare, Finland) were performed at day 0 (prior to wounding) and every 2–3 days for the following 56 days. Vascularization was quantified by converting each *in vivo* slit-lamp biomicroscopy image files to 8-bit via ImageJ and increasing the visualization of vasculature with the finding edges and contrast enhancement processes, along with inverting all black and white values. The converted images were then opened in GNU Image Manipulation Program (GIMP 2.8.20), a transparent overlay layer was added to each image file, vessels were manually traced on to the new layer, and finally, the area of traced vessels was converted to total pixel number using ImageJ (Fig. S3).

### Expression and re-establishment of immune tolerance by HLA-G in the injured cornea

Rabbits were euthanized and eyes were recovered and post fixed overnight, at 4 °C, in neutral buffered formalin and then stored in 70% EtOH until being paraffin embedded. Tissue was sectioned at 5 μM and histopathologically evaluated for the quantitation of cornea vasculature (CD31) and immune response looking at the expression of CD3, CD8, and CD4. Sections stained with hematoxylin and eosin were examined by light microscopy. The corneal histologic scoring scheme was modified from a previously described method^39^ and graded the following areas, degree of cellular infiltrate, extent of fibrosis, and extent of vascularization. Scores of 0–4 were assigned based on the following rubric: 0, no significant lesion; 1, low numbers of inflammatory cells, mild fibrosis, minimal vascularization; 2, moderate, diffuse stromal inflammatory cell infiltrates, fibrosis, or neovascularization; 3, moderate to marked diffuse stromal inflammatory cell infiltrate, fibrosis, or neovascularization; 4, marked, diffuse, cellular infiltrate, fibrosis, vascularization. Cumulative histologic scores were then analyzed and reported as mean ± standard deviation (SD) per treatment group.

### Immunohistochemistry

Cornea tissues were embedded in paraffin and using a microtome, 5 μm sections were cut and then mounted and dried onto slides. In order to stain the tissue sections and use Alexa Fluor conjugated secondary antibodies to image fluorescence, sections were deparaffinized and rehydrated. Deparaffinization was carried out by incubating slides twice, 10 mins each, in xylenes. Using a decreasing ethanol gradient, tissue was rehydrated by the slides being sequentially moved through two incubations in 100% ethanol for 3 minutes each, one incubation in 95%, one incubation in 80%, and finally, rinsed in deionized water for at least five minutes. Before staining tissue sections, heat-induced epitope retrieval (HEIR) procedure was performed at 98 °C for 15–20 minutes to unmask the epitopes of proteins that were crosslinked during the paraffinization process. Specifically, sections that were stained for HLA-G (Santa Cruz, sc-21799, dilution 1:50), GFP (Aves, gfp-1020, dilution 1:500), CD3 (Biorbyt, orb323391, 1:100), CD4 (Invitrogen, MA1-81588, 1:100), and CD8 (Novus Biologicals, NB10064021, 1:100) were immersed into pre-heated citrate based antigen retrieval solution, pH 6.0 (Vector Laboratories, H-3300), while the CD31 (Abcam, ab199012, 1 μg/ml) epitope was retrieved in 1 mM EDTA, pH 8.0, and the αSMA (Dako, M085101-2) epitope was retrieved in tris-based HEIR solution, pH 9.0 (Vector Laboratories, H-3301). After HEIR was completed, slides were allowed to cool for 20 minutes at room temperature and then sections were washed twice for 5 minutes each in TBS plus 0.025% Triton X-100 with gentle agitation. The non-specific binding sites within the tissues were then blocked by incubating the slides with 10% normal serum, tissues stained for HLA-G were blocked with normal horse serum while all others were blocked with normal goat serum, with 1% BSA in TBS for 1 hour at RT. Blocking solution was drained off of the slides and sections were then incubated overnight at 4 °C with primary antibody diluted appropriately in TBS with 1% BSA. After incubation, sections were washed two times, 5 minutes each, with TBS plus 0.025% Triton X-100 with gentle agitation to remove primary antibody that was non-specifically bound. A suitable fluorophore-conjugated secondary antibody was diluted according to manufacturer suggestions in TBS plus 1% BSA and was then added to each tissue section and incubated for 1 hour at room temperature with gentle agitation. Finally, slides were washed three times with TBS, five minutes each. Nuclei within each section were stained when coverslips were mounted with ProLong Diamond Antifade Mountant with DAPI (Molecular Probes, P36971). As an additional measure of fibrosis, Masson trichrome staining was done by the CGIBD Histology core at the University of North Carolina at Chapel Hill and the expression of profibrotic proteins for each treatment group was evaluated through microscopy. Images from each slide were taken using an Olympus IX83 research inverted microscope with a 40X objective (Olympus, Tokyo, Japan) and processed using Olympus cellSans life science imaging software.

### AAV8G9 Biodistribution following Stromal Injection

Serum was collected prior to the start and at the completion of these experiments and analyzed for neutralizing antibodies generated to the AAV capsid. Additionally, the liver, brain, kidney, heart, and draining lymph nodes were harvested for vector biodistribution assays using Q-PCR.

### Neutralizing Antibody Assay

The presence of neutralizing antibodies in rabbit serum was assayed using a previously described method with a couple of slight modifications^40^. HEK293 cells were seeded in 24 well plates at 5 × 10^4^ cells per well in 500 µl of complete DMEM (10% serum, 1% P/S). Cells were cultured for 24hrs at 37 °C and 5% CO_2_. Each rabbit’s pre- and post-injection serum was serially diluted, starting with twofold and going up to 1:256, in DPBS to a final volume of 12.5 µl. Serum dilutions were incubated with 3.84 × 10^8^ viral genomes (7,680 genomes/cell) of AAV8G9-Luciferase for 2hr at 4 °C. The seeded HEK293 cells were then transduced with virus-serum mixtures for 48hrs under optimal culture conditions before transduction efficiency was measured by a luciferase assay.

### Luciferase Assay

Luciferase assay conducted to measure the transduction efficiency of AAV8G9 in the presence of rabbit serum. Promega Luciferase Assay System was performed as follows. A 1X lysis reagent was prepared by diluting 5x Promega Passive Lysis Buffer in ddH_2_O. Media was aspirated and cells lysed by adding 250 µl of lysis reagent to each well of HEK293 cells and shaking the plate at room temp for 20 minutes. A mixture of 100 µl of lysed cells and 100 µl of Luciferase Assay Reagent was added to each well of a 96-well plate and using the Perkin Elmer Victor^3^ multilabel plate reader luminescence was measured as counts/second (CPS). Protein concentration was measured using a Nanodrop 2000c spectrophotometer and for each sample luciferase activity was normalized to the total protein concentration and reported as CPS/[P].

### Statistical Analysis

Data generated from the experiments in this study were analyzed with GraphPad Prism, version 6.0, and the specific test used for analysis was the unpaired T test. Significance was set at *p* < 0.05 for all comparisons in this study.

## Electronic supplementary material

Supplementary Figures
