# Supplementary material for: AAV vector-meditated expression of HLA-G reduces injury-induced corneal vascularization, immune cell infiltration, and fibrosis
Source: Sci Rep. 2017 Dec 19;7:17840. doi: 10.1038/s41598-017-18002-9 (PMC5736662; doi:10.1038/s41598-017-18002-9)
Supplement: Supplementary file 1 — Supplementary Figures [file 41598_2017_18002_MOESM1_ESM.pdf]

AAV vector-meditated expression of HLA-G reduces injury-induced corneal vascularization, immune cell infiltration, and fibrosis

Authors: Matthew L. Hirsch[1.2*], Laura M. Conatser[1.2*], Sara M. Smith[3], Jacklyn H. Salmon[3], Jerry Wu[1.2], Nicholas E. Buglak[1.2], Rich Davis[2], Brian C. Gilger[3]

Affiliations:

[1] Gene Therapy Center, University of North Carolina at Chapel Hill, NC, 27599, USA.

[2] Department of Ophthalmology, University of North Carolina, Chapel Hill, NC, 27599, USA.

[3] Department of Clinical Sciences, North Carolina State University, Raleigh, NC 27607, USA.

*These authors contributed equally to this work

Corresponding author:
Brian C. Gilger
North Carolina State University
1060 William Moore Drive
Raleigh, NC 27607
Phone 919-513-1273
Fax 919-513-6711
Email: bgilger@ncsu.edu

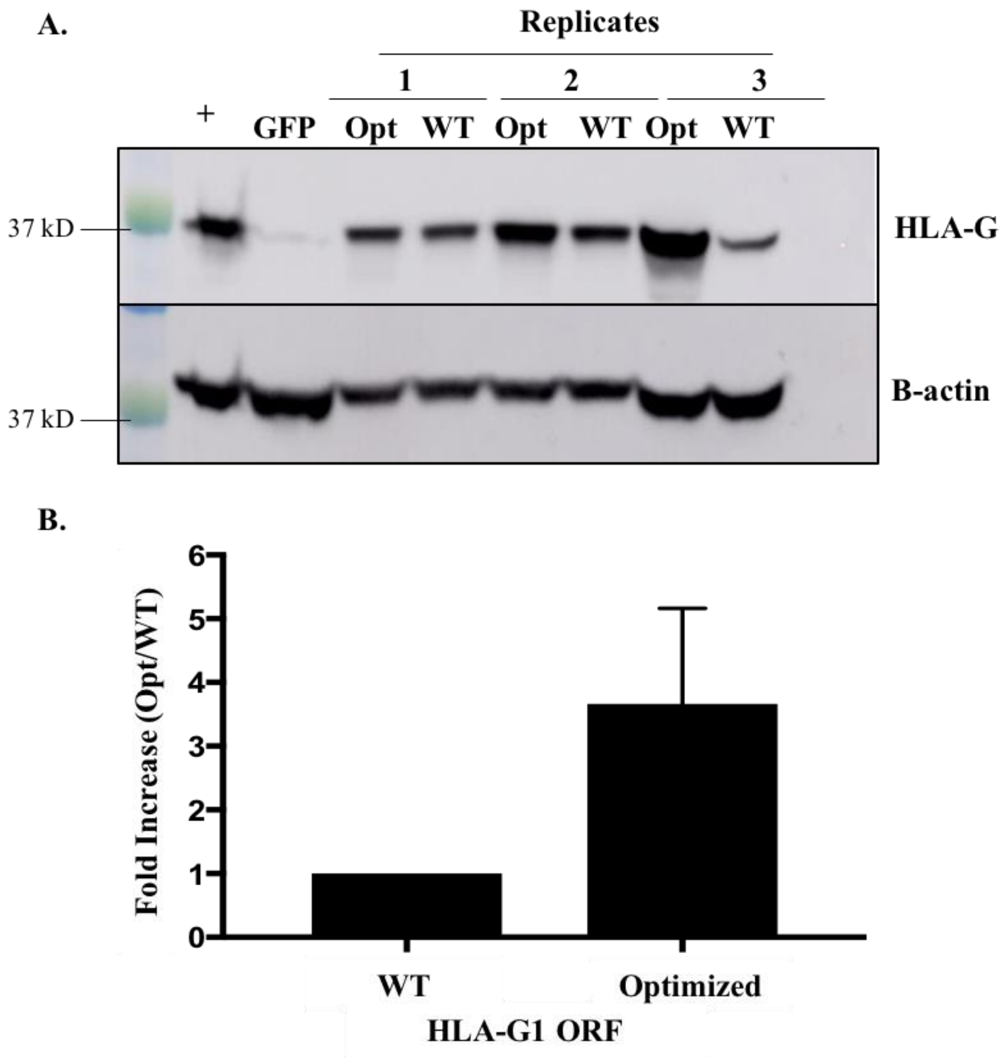

Supplementary Figure S1: Comparison of HLA-G Abundance from WT or Codon Optimized cDNA.
A) Identical plasmid contexts containing the WT HLA-G cDNA or codon optimized HLA-G cDNA were used in separate 293 cell transfections. Lysate was recovered 3 days post-transfection and analyzed for the indicated proteins by Western blot. B) Quantitation based of Western blot densitometry of the data depicted in (A). Opt = codon optimized, ORF = open reading frame

A.

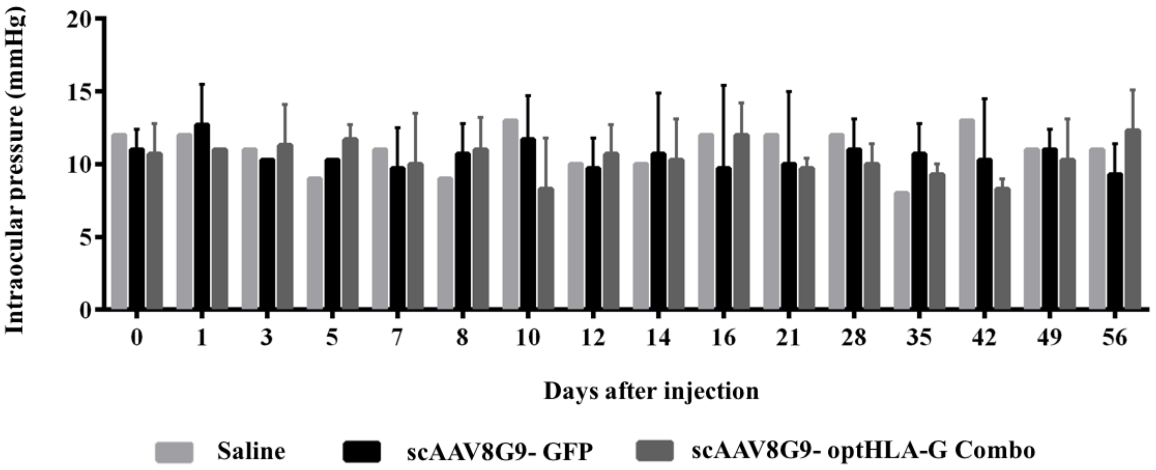

B.

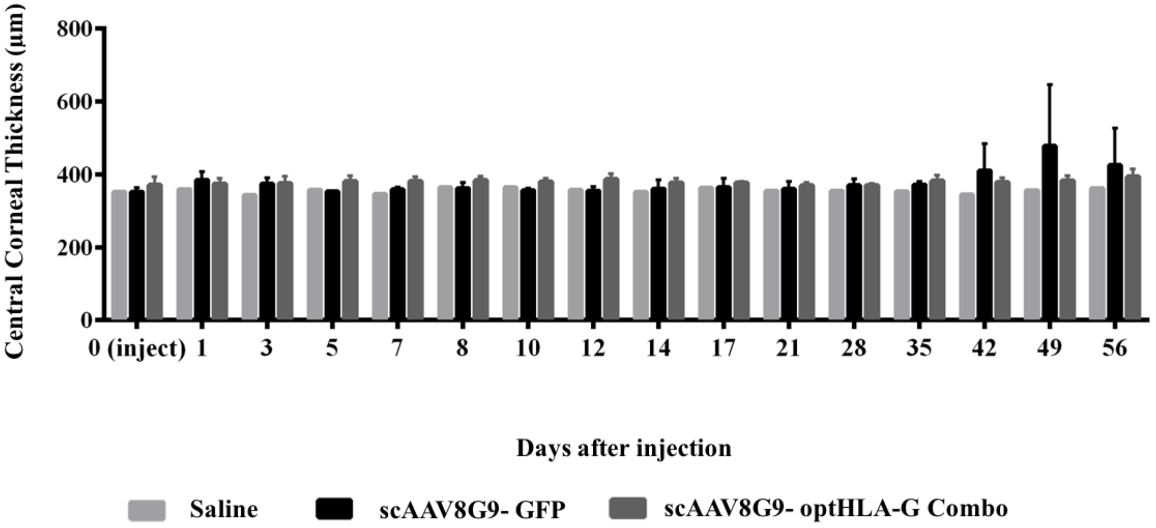

Supplementary Figure S2: Intraocular Pressure and Central Corneal Thickness Following AAV Vector Cornea Injections. Rabbit corneas were burned centrally eight days prior to a cornea intrastromal injection of saline, scAAV8G9-GFP, or scAAV8G9-optHLA-G Combo (isoforms 1 and 5 at a 1:1 ratio). A) Intraocular pressure is reported on the indicated day post-injection. B) Central corneal thickness is reported on the indicated day post-injection.

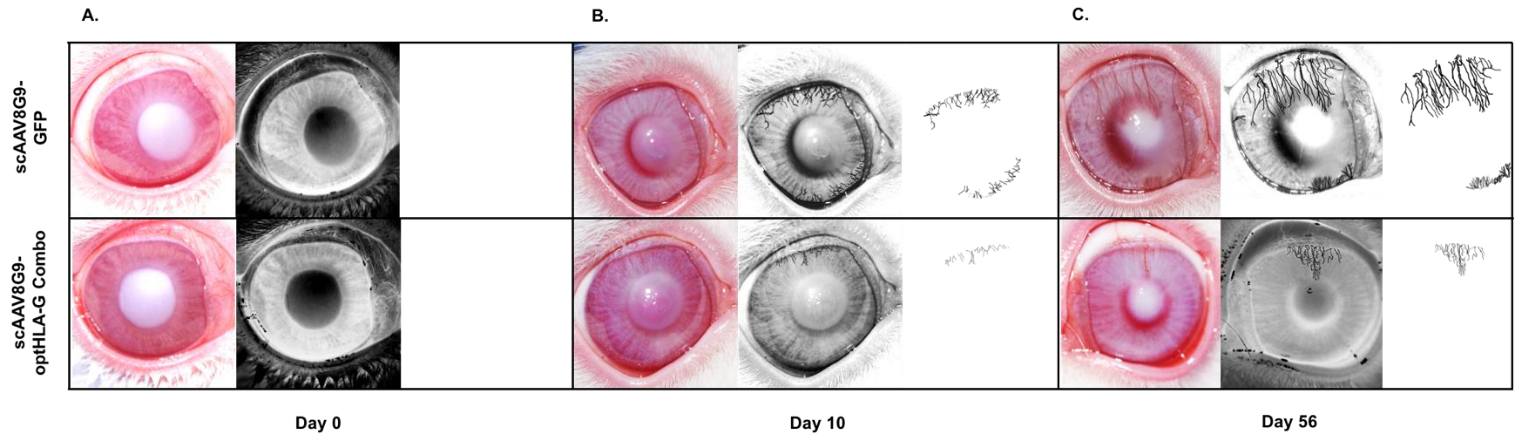

Supplementary Figure S3: Burn-induced Cornea Vascularization Analysis and Quantitation of *in vivo* Images. Centrally burned rabbit corneas were given an intrastromal injection of either scAAV8G9-GFP or scAAV8G9-optHLA-G Combo seven days after injury was induced. A-C) Representative images depicting traced vascularization from *in vivo* live images of treated corneas with the indicated vectors at the indicated time points to quantitate the total area of vessel ingrowth. sc = self-complementary

A.

Animal ID	Cellular Infiltrate	Fibrosis	Vascularization	Cumulative Score
**scAAV8G9-GFP**				
Rabbit-6	3	2	2	7
Rabbit-7	1	1	0	2
Rabbit-8	2	2	0	4
**scAAV8G9-optHLA-G Combo**				
Rabbit-12	0	0	0	0
Rabbit-13	0	1	0	1
Rabbit-14	0	0	0	0

Overall Histological Score		
	Mean	SD
scAAV8G9-GFP	4.33	2.52
scAAV8G9-optHLA-G Combo	0.33	0.58

B.

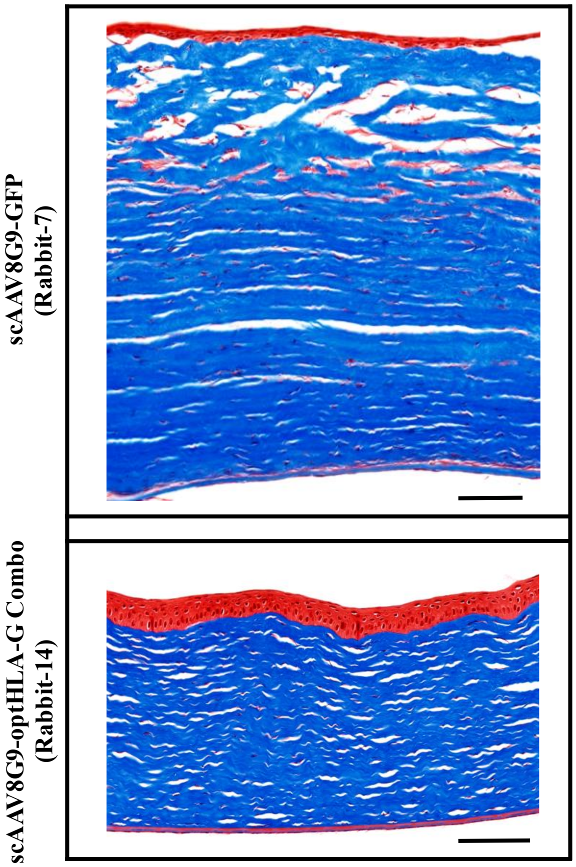

*Scale Bar = 100um

Supplementary Figure S4: scAAV8G9-optHLA-G Combo Inhibits Cornea Burn-induced Fibrosis. Trauma induced rabbit corneas acquired 60 days post treatment with the indicated vectors were each histologically evaluated and assigned a clinical score for cellular infiltrate, fibrosis, and the degree of vascularization (A). Masson trichrome stain (B) confirmed the presence of fibrotic fibers.

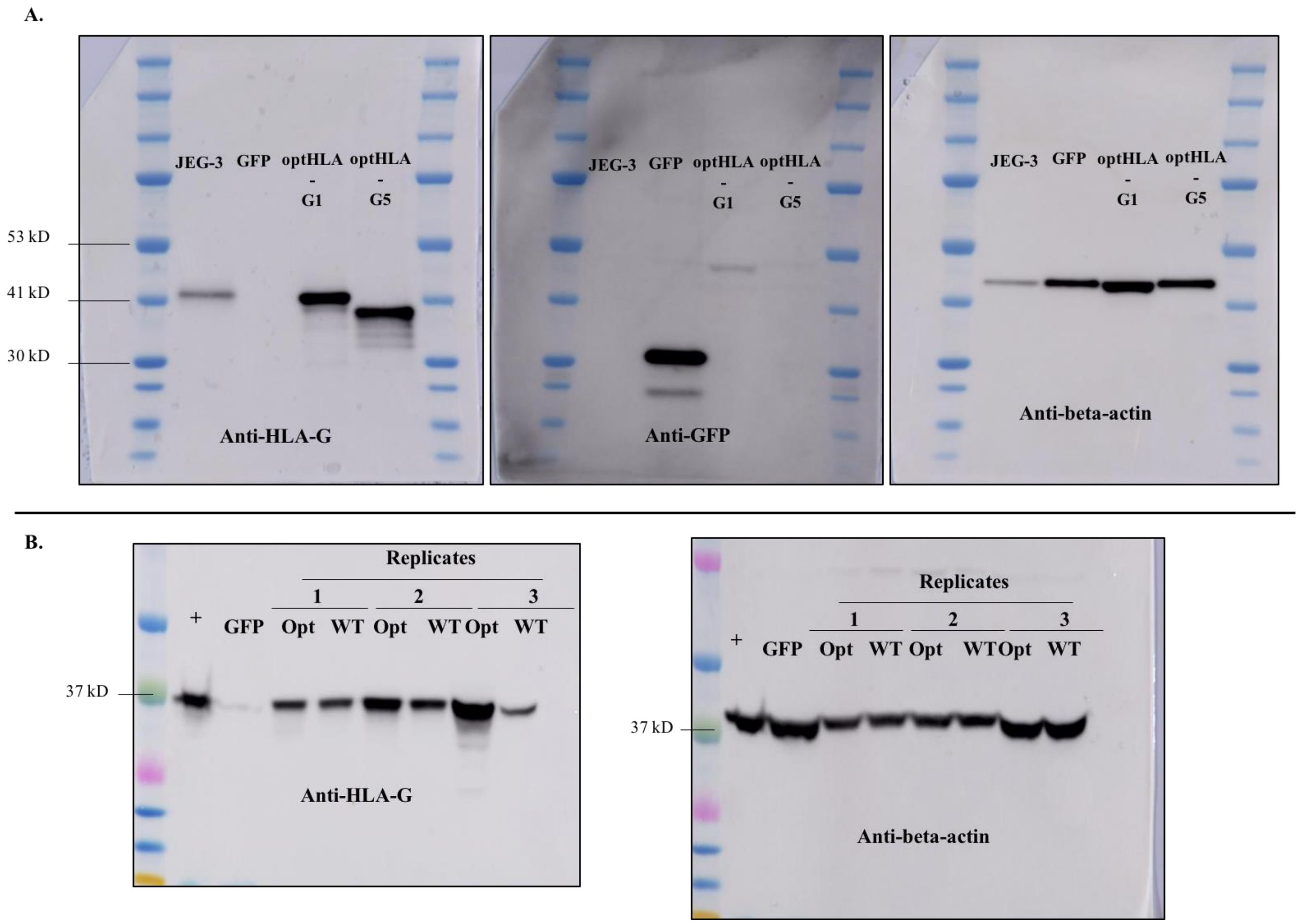

Supplementary Figure S5: Western Blot Analysis. A) Images of full-length blots shown in Fig. 1B, showing confirmation of optHLA-G isoform production in 293 cells by Western blot. JEG-3 cells are placental cells and serve as a positive control. B) Lysate recovered 3 days post transfection of identical plasmid contexts containing the WT HLA-G cDNA or codon optimized HLA-G cDNA were analyzed for the indicated proteins. Images of full-length blots depicted in Fig. S1.
